# Mechanical and Pore Properties of Concrete Using Basalt-Based Recycled Aggregates According to Mixture Conditions

**DOI:** 10.3390/ma15165677

**Published:** 2022-08-18

**Authors:** Hong-Beom Choi, Jin-O Park, Hyung-Do Lee, Kyeo-Re Lee

**Affiliations:** Construction Test & Assessment Center, Construction Test and Certification Department, Korea Institute of Civil Engineering and Building Technology, Goyang-si 10223, Korea

**Keywords:** basalt, recycled aggregate, concrete, cement content, fine aggregate

## Abstract

This study investigated recycled aggregates of Jeju Island—where porous basalt exists as a natural aggregate—and is a study aimed at verifying the applicability of the basalt-based recycled aggregate in the field. To this end, the application properties of concrete were analyzed using the quality improvement of the recycled aggregate (PRA), the type of fine aggregate received in the region, and the cement content as variables. In an experiment using concrete in which 50% of the PRA was replaced with basalt (BA), the air content of the fresh concrete decreased due to the increasing solid content of the aggregate when PRA and fine aggregates (with an even particle size distribution) were used. Regarding the properties of the hardened concrete, when the PRA and fine aggregate (with a high fineness modulus) were used, the compressive strength was 33.6 MPa and the modulus of elasticity was 23.1 GPa, which are higher than those of the BA specimen. The resistance to carbonation increased due to the improved quality of the PRA specimen. Pores that are 0.3 mm in size or larger decreased when the PRA and fine aggregates of even particle sizes were used. This form of pore reduction was found to have a direct correlation with the improvement of mechanical properties.

## 1. Introduction

Large-scale capital development projects on Jeju Island, including the construction of Jeju’s second airport and its new port, are scheduled, all of which require enormous volumes of construction materials, such as cement and aggregate [1]. Consequently, the number of quarries increased due to the construction boom on Jeju Island over the last years. As a result, environmental damage on Jeju Island accelerated, and it is uncertain whether quarry permits will be extended in the future. Hence, it is expected that the supply of aggregates necessary for the Jeju Island development projects may become more difficult [2,3]. Recently, recycled aggregate concrete has been successfully applied to every floor of a 20-story building in South Korea, and recycled aggregate use in construction work is increasingly emerging as a solution to the current supply shortage [4]. However, most recycled aggregates produced on Jeju Island are made of porous basalt with a high absorption rate and have not yet received quality certification for use in concrete because the technology for removing cement paste and mortar remains inadequate [5,6].

The abrasion rate, absorption rate, and density of recycled aggregate can affect concrete quality, as discussed in the literature. Various studies on their physical, chemical, and curing properties have been proposed to improve the quality of recycled aggregates. Typically, such researched physical methods aimed at impacting old cement paste using crushing and grinding equipment, with chemical methods, such as removing old cement paste using strong acid solutions, suggested. Unlike the above two methods, the curing method reinforces the matrix by transforming Ca(OH)_2_ to CaCO_3_ by injecting CO_2_ into recycled aggregate containing old cement paste. Various methods for enhancing the quality of recycled aggregates by applying or combining these properties are being studied [7,8,9,10,11,12,13,14,15,16]. The research results may vary depending on the quality and production method of recycled aggregates. Several studies have reported that less than 30% of recycled aggregates have a harmful effect on concrete [17,18]. In addition to this, it is reported that concrete with a strength of less than 30 MPa has a significantly low impact on the use of recycled aggregates [19]. The research to improve the quality of recycled aggregate concrete reports the effect of supplementary cementitious materials (SCMs) in consideration of strong alkalinity caused by old paste [20,21]. The impact of the improvement in the durability of recycled aggregate concrete is reported by using fibers and adjusting the curing conditions [22,23]. The effects of the properties of the parent rock included in the recycled aggregate on the recycled aggregate concrete have also been researched extensively [24,25,26]. However, there has been insufficient research on recycled aggregates containing Jeju basalt, which has different properties from overseas basalts and domestic and overseas quartz aggregates, which are used in high-strength concrete owing to their low porosity. The considered reason that recycled aggregates on Jeju Island are difficult to use is the high absorption rate due to the high porosity of Jeju basalt, for which its range of absorption rate is 0–10% [27,28].

In a recent study on recycled Jeju aggregates, a recycled aggregate with an absorption rate of 3–5% was produced using a physical method that considered time and costs, in which its concrete properties of mix strength (50 MPa) and performance (being equal to or higher than that of general Jeju basalt) were verified [29]. However, the recycled aggregate produced by this research study exhibited a low coarse-aggregate recovery rate due to the repeated process of quality improvement. The research results also showed that it did not have a meaningful effect on the concrete, even if the quality of the recycled aggregate was somewhat low. Therefore, as a basic study to apply Jeju Island’s recycled aggregates to buildings and to commercialize them, this study examined the general strength properties of recycled-aggregate-based concrete by improving the aggregate recovery rate to mitigate the problems inherent in previous studies. Furthermore, considering the realities of Jeju Island—where it can be difficult to produce fine aggregates—this study analyzed the application characteristics of imported sea sand and crushed sand and the effect of recycled aggregate concrete on changes in the unit’s cement content. By conducting this study, the characteristics of concrete mixing and the dynamic relationship between aggregates were analyzed, the results of which could be used as basic data for resource recycling and for methods that use them based on quality.

## 2. Experimental Methods

### 2.1. Experimental Plan

This study examined mixes of fine aggregates and binders for the practical use of recycled aggregates for concrete on Jeju Island. Table 1 outlines the experimental plan. Experimental variables were coarse and fine aggregate types and unit cement content. The coarse aggregates include mixtures of basalt with natural basalt aggregate (BA), raw recycled aggregate (RRA), and improved recycled aggregate (PRA), respectively. Fine aggregates include sea sand (S), crushed sand (C), and fine aggregate mixed with sea sand and crushed sand in a 4:6 ratio (SC). The unit cement contents of these three types of aggregates are 300, 330, and 360 kg/m^3^, respectively. For these three variables and three factors, nine mix proportions were selected using the Taguchi method of experimental planning, the selected mix proportions of which are listed in Table 2. The water–cement ratio and the fine aggregate ratio are fixed at 50% and 40%, respectively. It was assumed that the old paste of RRA and PRA, which are recycled aggregates, did not react in a pozzolanic manner because they were non-reactive substances. We only considered the change in the amount of cement paste due to the change in cement content.

### 2.2. Experimental Materials

**Figure 1:**
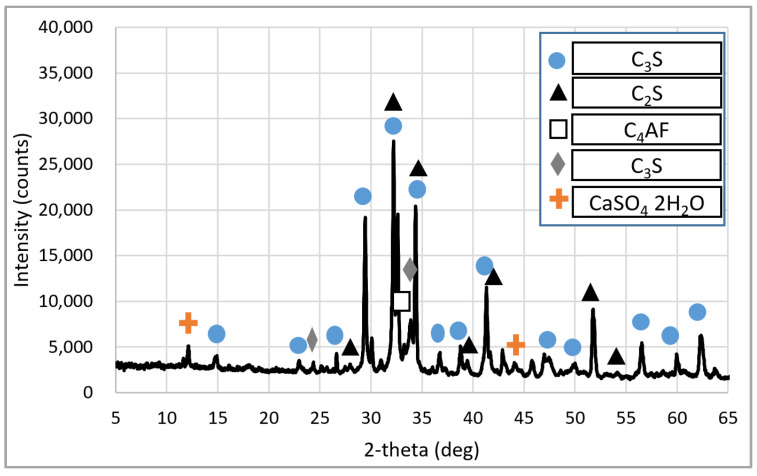
Mineral composition of cement.

**Figure 2:**
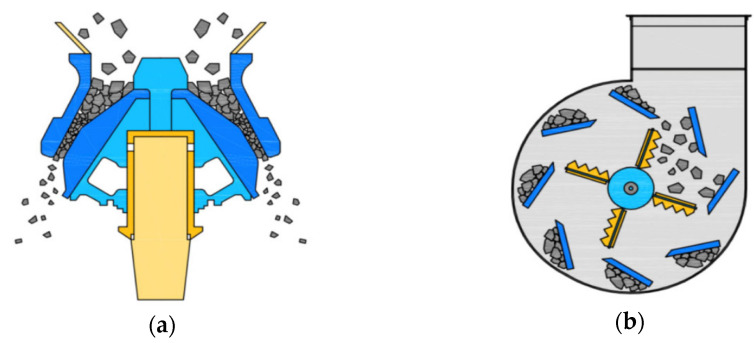
Manufacturing process of the recycled aggregate. (**a**) Type A; (**b**) Type B.

**Figure 3:**
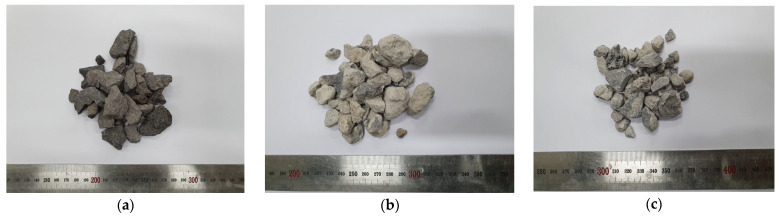
Using the coarse aggregate. (**a**) BA; (**b**) RRA; (**c**) PRA.

**Figure 4:**
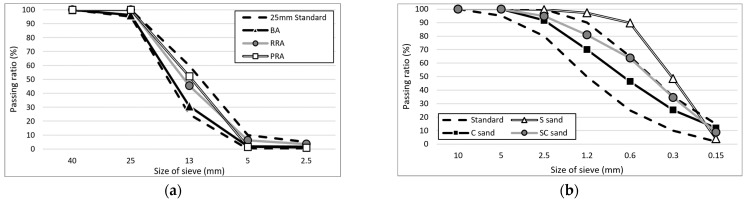
Grading of the aggregate used. (**a**) Coarse aggregate; (**b**) Fine aggregate.

The cement used in the experiment was ordinary Type 1 Portland cement from ‘S’ Company (Dangjin City, Korea). The physical and chemical properties of the cement are shown in Table 3 and Table 4, respectively, and the mineral composition of cement is shown in Figure 1. The cement used satisfies the standard conditions for Type I Portland cement of ASTM C150:2020 [30]. BA (the Jeju Island natural aggregate) was an aggregate from Namwon-eup, Seogwipo-si. It is a 5–25 mm aggregate that satisfies the standard conditions for concrete aggregate in ASTM C33:2018 [31]. The recycled aggregate was produced using the Type A and Type B methods shown in Figure 2. Type A is a method of crushing aggregates by transmitting an impact force to the entire aggregate. The principle is that the mantle that is connected to the central axis rotates without eccentricity and crashes with the aggregate between the top wall and the mantle, whereas Type B is a method in which aggregates are first sent upwards via a bucket before being dropped onto a high-speed rotating blade; the aggregates are produced to peel the old paste by applying an impact to the surface of the aggregate using the blade. The recycled RRA and PRA were produced for the sub-base using the Type A method, after which the PRA was improved using Type B as a secondary process. An image of the coarse aggregate is shown in Figure 3, and the physical properties are listed in Table 5. The particle size curves of each aggregate are shown in Figure 4. The RRA and PRA were mixed with BA. The chemical compositions of the aggregates are listed in Table 6.

### 2.3. Experimental Methods

**Figure 5:**
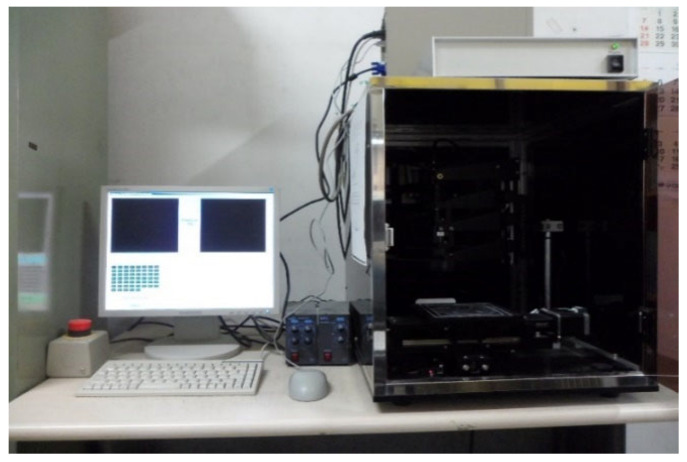
Image analysis device (HF-MA C01).

We conducted experiments in accordance with ASTM standards to analyze the fresh properties and hardened properties of the concrete. Accordingly, a slump of the fresh concrete was measured using a slump cone with a base diameter of 200 mm, top diameter of 100 mm, and height of 300 mm based on ASTM C143:2020 [32] specifications. It was further divided into three parts and was determined using a pounding bar. Then, the slump cone was slowly lifted for the purpose of conducting measurements. The air content of the fresh concrete was measured using a Type-B air meter with capacity of a 7.0 L based on ASTM C231:2017 [33] specifications. After being determined based on the division into three equal parts with a pounding bar, the volume of air was measured using a water-injection method. The compressive strengths of cylindrical concrete specimens of 100 mm diameter and 200 mm height were then determined using a stress rate of 0.25 MPa/s for three or more specimens aged for 3, 7, 28 d, and 1 year. To prevent eccentricity, the concrete specimen to be measured was subjected to an equal distribution load by capping the upper surface with a paste with a strength higher than that of the high-strength concrete. The samples were measured in accordance with the ASTM C39:2020 [34] standard. The flexural strengths of cuboid concrete specimens of dimensions 100 × 100 × 400 mm were determined using a loading rate of 1.0 MPa/min for three or more specimens of 28 d age, in accordance with the ASTM C78:2018 [35] standard. The equipment used for compressive strength and bending strength is UTM (Universal Testing Machine) (HCT-DC 100, “H” company, Gimpo, Korea). The modulus of elasticity was measured using a loading rate of 0.25 MPa/s for three or more specimens by attaching strain gauges (PL-60-11, “T” company, Tokyo, Japan) to the 28-day-old cylindrical specimens. The samples were measured by applying a load in accordance with the ASTM C469:2014 [36] standard (concrete cylindrical specimen; static modulus of elasticity) and the Poisson’s ratio test method. At this time, the data were collected using the data logger (TDS-540, “T” company, Tokyo, Japan) according to the strain and stress. The accelerated carbonation experiment was performed in accordance with RILEM CPC 18 recommendations using cylinder concrete specimens of diameter 100 mm and height 50 mm. The accelerated carbonation conditions were created using a CO_2_ concentration of 5 ± 0.2% [37]. The concrete specimens were split by UTM after 1, 4, 8, 13, and 26 weeks of age, and the depth to the discolored section is measured by spraying a phenolphthalein solution on the split surface. The pore structure of the hardened concrete was analyzed using the linear movement method and the improved pore-counting method as specified in the ASTM C457 Standard Test Method for Microscopical Determination of Parameter of the Air-Void System in Hardened Concrete. The pore’s structure was analyzed using an image analyzer (HF-MA C01, “F” company, Yamato, Japan), which uses an optical microscope, as shown in Figure 5 [38]. The experiment considered a cylinder-shaped concrete specimen with a diameter of 100 mm and a height of 50 mm. A surface polishing machine (HS-1440B, “H” company, Gimpo, Korea) with a diamond disc plate was then used to level the surface in order to distinguish the void from the surface. For polishing, 120-, 400-, and 600-grit surfaces of the diamond disc plate were used. The image analyzer was used to measure the pores on the flat surface of the concrete specimen. In multiple regression analyses, all of the different combinations of the variables used were input at the same time to distinguish between significant and non-significant variables. The variables were then analyzed based on their ability to estimate information regarding the different specimens. As shown in Table 7, the independent variables were water, cement, BA, RRA, PRA, S sand, and C sand. In correlation analyses, the correlation between the materials and mechanical properties of the concrete according to the change in the formulation was analyzed using Pearson’s correlation analysis.

## 3. Results and Discussion

### 3.1. Slump

**Figure 6:**
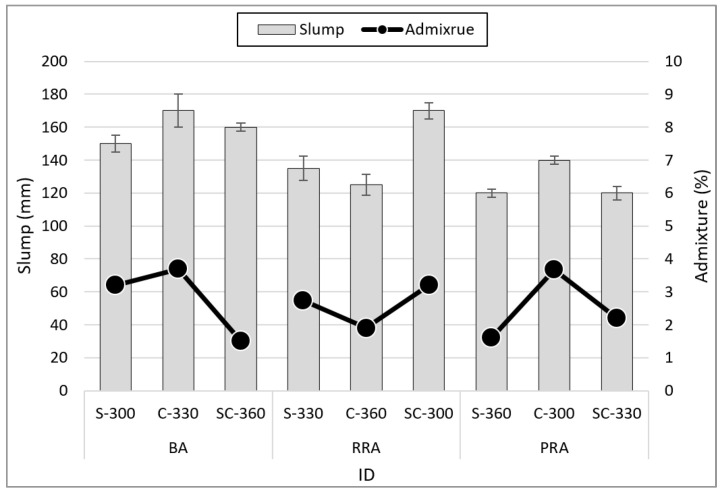
Slump of fresh concrete.

Figure 6 shows the slump results based on the mix conditions of non-hardened concrete using the recycled Jeju aggregate, with slump measurements ranging from 120 to 170 mm. Among the types of fine aggregate, the formulation that included C fine aggregate required the largest volume of high-performance water-reducing agents to satisfy the target range of the slump. This is due to the increase in the specific surface area of the fine aggregate C because of the relatively large number of fine aggregates smaller than 0.15 mm. Conversely, an increase in unit cement content can be correlated with a decrease in the volume of high-performance water-reducing agents. This is a result of the increase in unit cement content that leads to an increase in unit water volume because of the fixed water–cement ratio. The slump appears to decrease as the recycled aggregate is used. However, the slump is not reduced by recycled aggregate because the volume of high-performance water-reducing agents is also reduced.

### 3.2. Air Content

**Figure 7:**
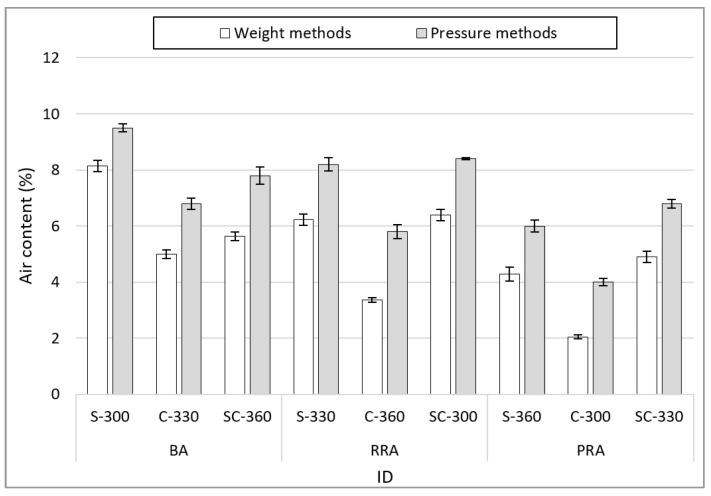
Air content of fresh concrete.

Figure 7 shows the results of the air content based on the mix conditions of non-hardened concrete using the recycled Jeju aggregate. The air content was measured using mass and pressure methods. The average difference in the air content between the two measurement methods for each aggregate type is 1.78% for BA, 2.13% for RRA, and 1.86% for PRA. It can be considered that there is a difference in proportion to the absorption rate because moisture penetrates into the aggregate under pressure because a porous aggregate was used, although pre-wetting was performed when using it. The air content by aggregate type is the highest in BA, followed by RRA and PRA. It can be surmised that this is because the concrete’s fillability increases due to the increased solid content in the aggregate when BA and recycled aggregate are mixed. In particular, it can be surmised that the effect is greater in the case of PRA due to the reduction in water absorption and the increase in solid content caused by the improved aggregate shape and the removal of old paste through the quality improvement process. By fine aggregate types, S exhibits the largest air content, followed by SC and then C. This can be considered to be because the proportion of aggregate in the same volume increases due to the even particle size distribution of C, similarly to the effect of coarse aggregates.

### 3.3. Compressive Strength

**Figure 8:**
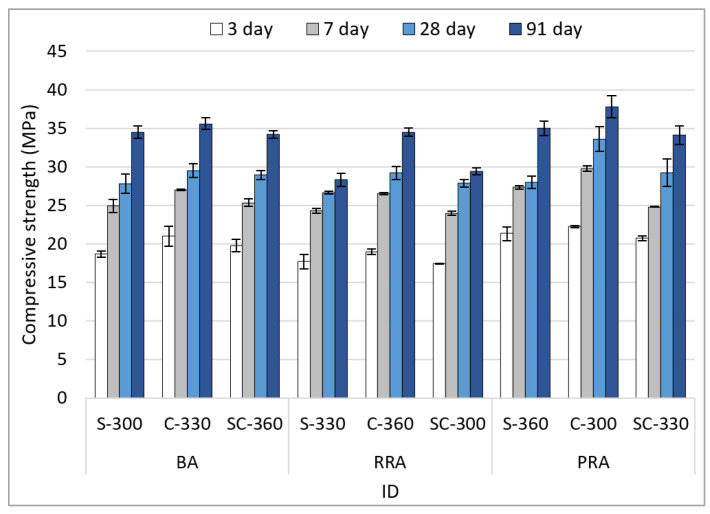
Compressive strength of the concrete.

Figure 8 shows the compressive strength results based on the mix conditions of concrete using the recycled Jeju aggregate. There was no significant difference in the compressive strength according to age. For the cement of age 28 days, the recycled aggregate with high water absorption (such as RRA) was used in the general-strength concrete with a mix strength of 26 MPa. However, there is no decrease in compressive strength due to the old paste. Among the PRA specimens, PRA-C-300 exhibits the highest compressive strength (33.6 MPa). This is because the fillability of the concrete improves as the sphericity of the aggregate increases as the old paste is removed. For the cement of age 91 days, PRA-C-300 showed the highest compressive strength of 37.8 MPA, and although the strength increased to a 40 MPa level, there was no decrease in strength due to the use of recycled aggregates. However, in the case of concrete using RRA aggregates, the compressive strength decreased from the initial days of aging. This could be because of the vulnerability induced by the large amounts of old paste contained in the RRA aggregate. In addition, since SCMs were not used in the experiment, there is no reinforcement of the cement matrix due to the components discharged by the old face, possibly contributing to the decrease in compressive strength.

**Figure 9:**
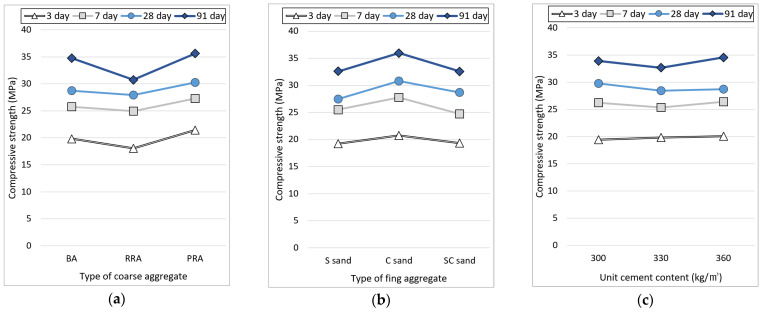
Compressive strength of the concrete depending on mixture conditions. (**a**) Coarse aggregate; (**b**) Fine aggregate; (**c**) Unit cement content.

Figure 9 shows the compressive strength results for each condition. As mentioned above, there is no decrease in strength owing to the use of the recycled aggregate based on the strength for 3, 7 and 28 days. However, in the case of RRA, the strength increase rate is lower compared to other aggregates; thus, it is judged that the compressive strength due to various old pastes is affected for the cement aged 91 days. In the case of the fine aggregate, the compressive strength is high when the fine aggregate C of high fineness modulus is used. This can be considered to be because the volume filled with the aggregate increases due to the even particle size of the fine aggregate, and the number of pores that could affect the compressive strength decreases. The compressive strength does not increase due to an increase in unit cement content. This is due to the increase in mixing water along with the cement’s content.

### 3.4. Flexural Strength

**Figure 10:**
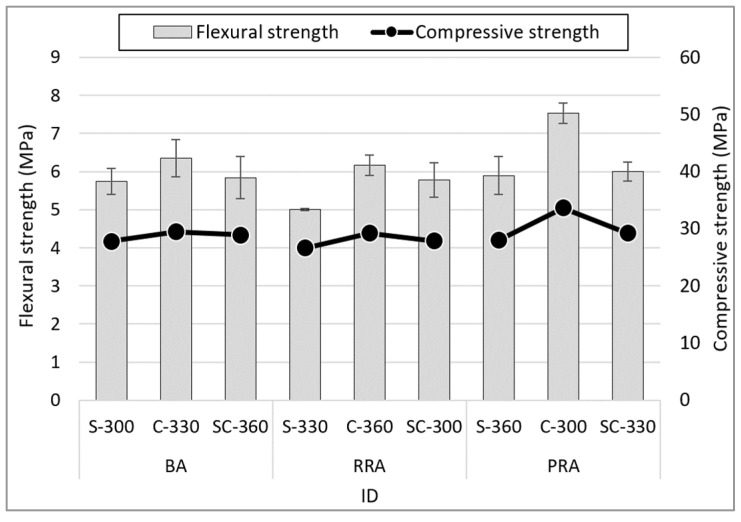
Flexural strength of the concrete.

Figure 10 shows the 28-day age flexural strength based on the mix proportion of concrete using the recycled Jeju aggregate. It appears that the unit cement content does not meaningfully affect the effect of flexural strength, although some changes in flexural strength by type of aggregate can be observed. For example, when using PRA, the concrete’s fillability due to the processing of the aggregate shape does appear to affect the flexural strength. Moreover, it can be considered that the type of fine aggregate has the greatest influence on the flexural strength of the concrete. PRA-C-300 using C fine aggregate exhibits the highest flexural strength (7.53 MPa). Other specimens using coarse aggregate exhibit relatively high strengths when C fine aggregate is used. The trend is similar when comparing compressive strength and flexural strength based on 28 days of aging. However, using fine aggregate C exhibits higher flexural strength than when using other fine aggregates. The compressive strength increases by 12.0% when fine aggregate C is used compared to when fine aggregate S is used. However, the flexural strength increases by 20.4%. This can be considered to be because the S fine aggregate homogenizes the concrete with an even distribution of fine particles less than 0.15 mm to relatively large aggregates of 2.5 mm [39,40,41].

### 3.5. Modulus of Elasticity

**Figure 11:**
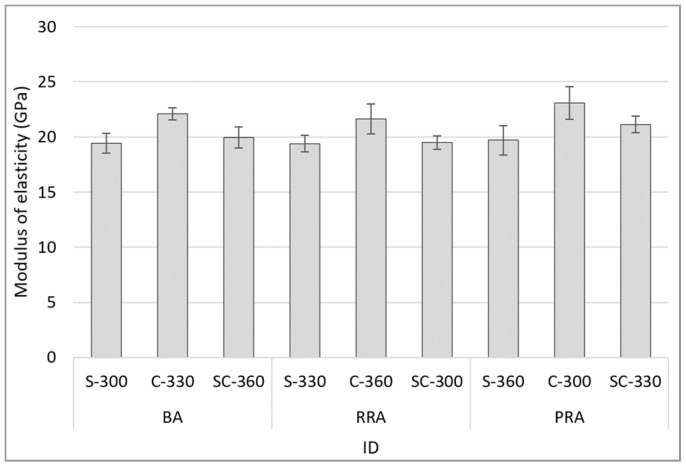
Modulus of elasticity of concrete.

Figure 11 shows the modulus of elasticity at 28 days of age based on the mixing of concrete using the recycled Jeju aggregate. The modulus of elasticity based on the use of recycled aggregates does not exhibit a substantial decrease, and no tendency based on the unit cement content can be observed. However, specimens using fine aggregate C exhibit a high modulus of elasticity based on the fine aggregate type. In particular, PRA-C-300 using PRA with good aggregate sphericity exhibits the highest modulus of elasticity at 23.1 GPa. It can be considered that this is due to improvements in the mechanical properties as the concrete’s fillability improved when using coarse and fine aggregates.

**Figure 12:**
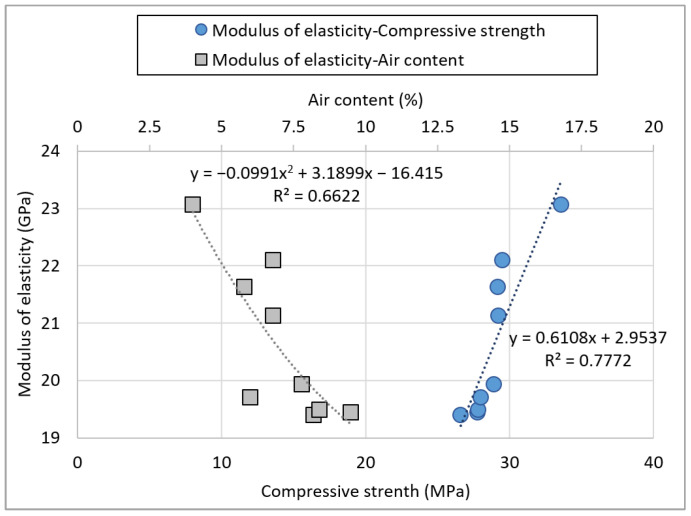
Effect of the modulus of elasticity on compressive strength and air content.

Figure 12 shows the relationship between the compressive strength and the air content of non-hardened concrete as a variable influencing the modulus of elasticity. Most studies on recycled aggregates in the basalt and quartz aggregate areas have reported that the compressive strength and modulus of elasticity showed a proportional relationship with one another [42,43,44,45,46]. The relationship between the modulus of elasticity and compressive strength in this experiment exhibits a similarly high tendency.

The relation equation of compressive strength and modulus of elasticity of basalt-based recycled aggregate concrete in Jeju Island is as follows:

(1) y=0.6108x+2.9537

where ***x*** represents the compressive strength, and ***y*** describes the modulus of elasticity of concrete.

However, there is no decrease in compressive strength and modulus of elasticity with an increase in the replacement rate of recycled aggregate mentioned in many studies. It is known that mechanical performances deteriorate when recycled aggregates containing a relatively large number of pores are used. However, the results of this study suggest that the increase in the concrete’s fillability of the concrete owing to the improvement of the aggregate shape has a greater effect. Moreover, it can be considered that the weak part of the old paste does not act at the general strength of 30 MPa. In addition, the increase in pores due to the type of fine aggregate appears to have a greater effect.

The relation equation of the modulus of elasticity and air content of basalt-based recycled aggregate concrete in Jeju Island is as follows:

(2) $$y=-0.0485x^{2}-1.3273x+27.469$$

where ***x*** and ***y*** represent the air content of fresh concrete and the modulus of elasticity of hardened concrete, respectively.

The compressive strength does not exhibit any correlation with the air content in non-hardened concrete. However, the modulus of elasticity decreases as the air content increases, indicating an inversely proportional relationship. This suggests that the modulus of elasticity could be more directly affected by the concrete pores than its compressive strength.

### 3.6. Depth of Accelerated Carbonation

**Figure 13:**
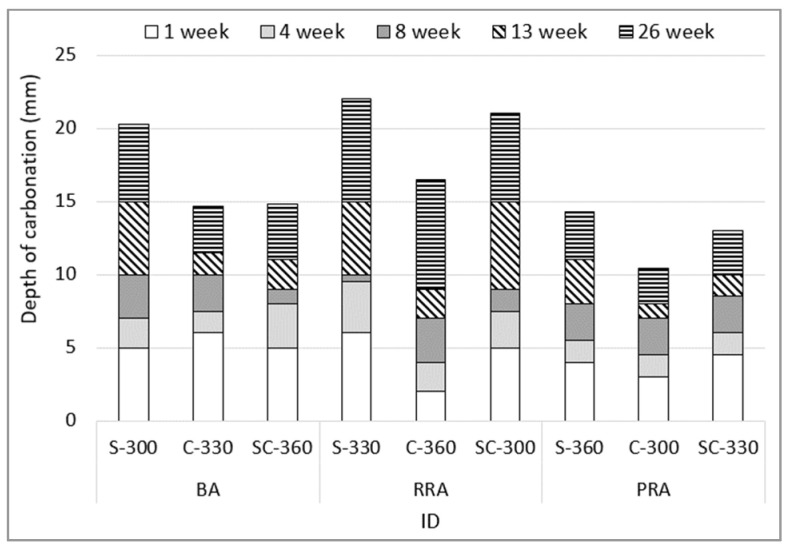
Depth of the accelerated carbonation of the concrete.

Figure 13 shows the depth of carbonation based on the mix proportion of concrete using the recycled Jeju aggregate. By the type of recycled aggregate, the depth of carbonation decreases based on the use of improved PRA. Many studies on recycled aggregates reported that an increase in the replacement rate of recycled aggregate increased the depth of carbonation of concrete [47,48]. However, the PRA specimen exhibits a different tendency from the conventional because it forms dense concrete due to the improved aggregate shape. BA and RRA exhibit similar depths of carbonation initially, but the depth of carbonation increases sharply after 13 weeks. It can be considered that the old paste of RRA with an alkali component is neutralized by the continuous CO_2_ supply, with the porosity of the old paste acting as a weak part for carbonation.

**Figure 14:**
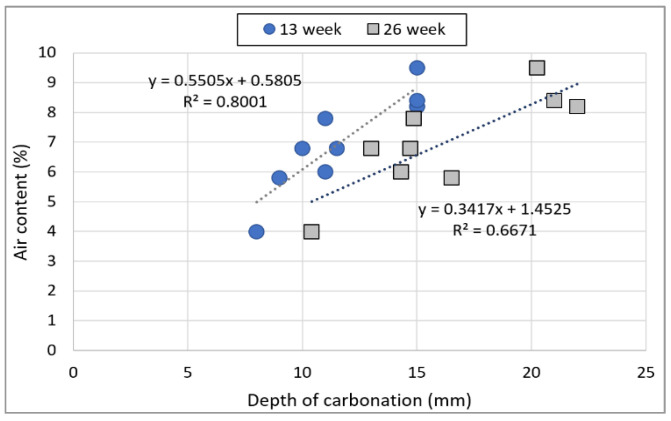
Relationship between the depth of carbonation and air content.

Figure 14 shows the relationship between the air content of non-hardened concrete and the depth of carbonation of hardened concrete. Dense concrete is generally known to be resistant to carbonation. Furthermore, the air content and depth of carbonation exhibit a proportional relationship with one another. The R^2^ value of the relationship between the depth of carbonation and air content after 13 weeks is as high as 0.80, and the correlation of the air content with the depth of carbonation decreases marginally after 26 weeks. This can be considered to be because the carbonation resistance decreases as the old paste of RRA is neutralized. It can be surmised that if the recycled aggregate contains a large volume of old paste, carbonation resistance will decrease as the age increases.

### 3.7. Pore Properties

**Figure 15:**
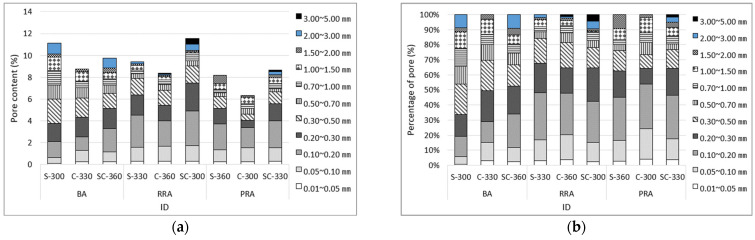
Pore content and percentage in concrete mixture. (**a**) Pore content in concrete; (**b**) percentage of pore distribution in concrete.

**Figure 16:**
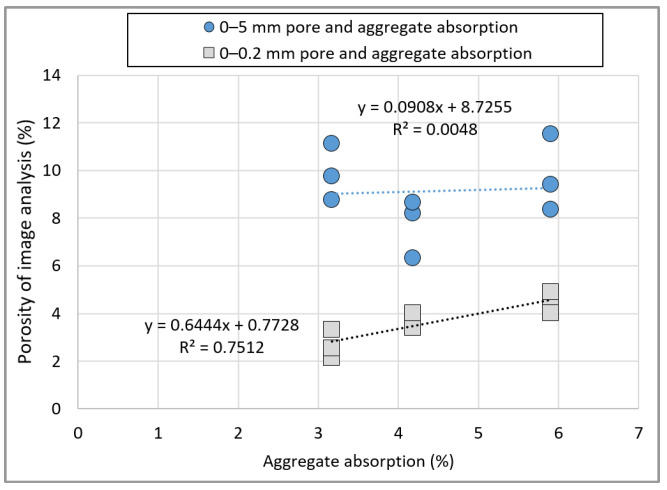
Relationship between the aggregate absorption and porosity.

**Figure 17:**
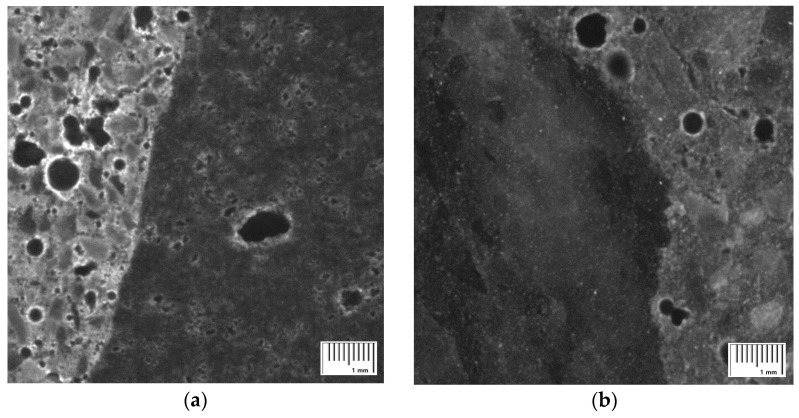
Optical microscope images of concrete. (**a**) Pore of basalt aggregate concrete. (**b**) Pore of quartz aggregate concrete.

Figure 15 shows the image analysis results of hardened concrete based on the mix proportion of concrete using the recycled Jeju aggregate, and the results also include all pores in the aggregate. The PRA specimen exhibits the smallest number of pores based on the total range of pores. Among them, pores smaller than 0.2 mm account for more than 50% of the total. The BA specimen exhibits the smallest content of pores smaller than 0.2 mm, whereas the RRA specimen exhibits the largest. This trend is similar to that of the aggregate absorption rate, as shown by the relationship between the aggregate absorption rate and porosity (Figure 16.). No relationship can be observed between the pores and aggregate absorption in the range of 0–5 mm. However, the relationship between pores in the range of 0–0.2 mm and aggregate absorption is high, with an R^2^ value of 0.75. It can be considered that the ratio of 0–0.2 mm pores due to the aggregate would be high, and the image shown in Figure 17 indicates the existence of large and small pores due to pores in the basalt.

**Figure 18:**
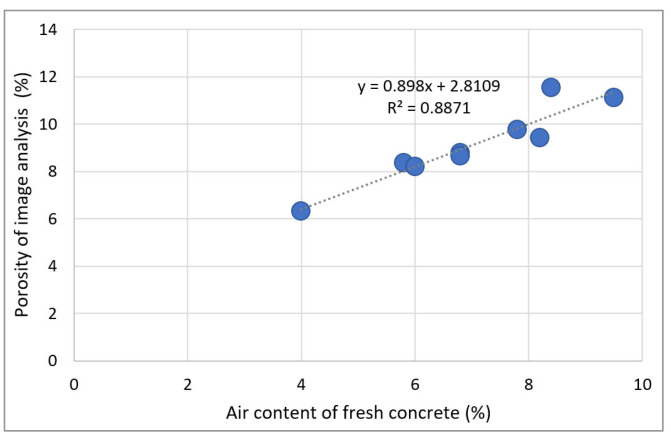
Relationship between the air content of fresh concrete and porosity of hardened concrete.

Figure 18 shows the relationship between the air content of fresh concrete and the porosity of hardened concrete.

The relation of porosity and air content of basalt-based recycled aggregate concrete in Jeju Island is described as follows:

(3) y=0.8871x+0.8871

where ***x*** and ***y*** represent the air content of fresh concrete and the porosity of image analysis of hardened concrete, respectively.

The air content of fresh concrete causes the collapse of trapped pores before hardening, thus decreasing the pore content of the hardened concrete. However, the main aggregate used in this experiment is basalt, and the air content partially increases because a large number of pores (large and small) are included in the aggregate. The correlation between the air content of the unhardened concrete and the porosity of the hardened concrete is high, with an R^2^ value of 0.889. Consequently, the pore-related characteristics—such as the unconsolidated air content, depth of carbonation, and modulus of elasticity—exhibit similar tendencies.

**Figure 19:**
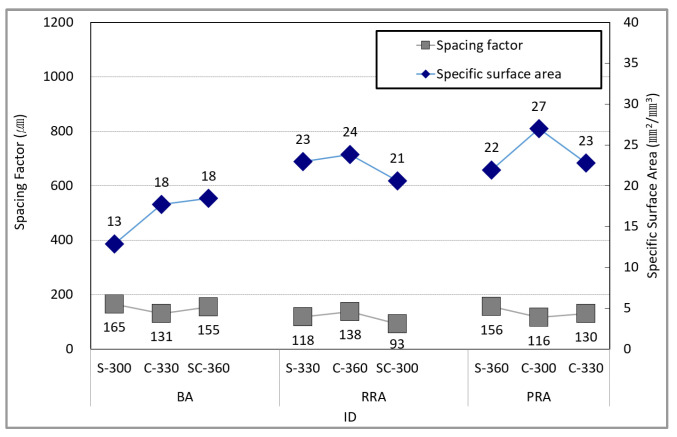
Spacing factor and specific surface area of the concrete.

Figure 19 shows the relationship between the pore-spacing factor and pore-specific surface area of concrete. In their book *Concrete*, Mindess et al. suggested that in order to reliably prevent freeze–thaw, the specific surface area of pores should be at least 25 mm^2^/mm^3^ and the spacing factor should be less than 200 μm. Furthermore, the Kansas DOT claimed that the spacing factor for concrete before hardening should be 250 μm or less [49]. In this study, the spacing factor satisfies the condition of 200 μm in all formulations, indicating resistance to freeze–thaw. However, in the case of the specific surface area of the pores, only the PRA-C-300 specimen can be found to exceed 25 mm^2^/mm^3^. In the case of the formulation containing RRA and PRA, the specific surface area of the pores can be found to approach 25 mm^2^/mm^3^ owing to the influence of the old paste. Consequently, it can be expected to have a relatively high resistance to freeze–thaw.

### 3.8. Multiple Regression and Correlation Analysis

A multiple regression analysis was conducted, as shown in Table 8, to verify the effect of water, cement, BA, RRA, PRA, S sand, and C sand on the carbonation depth. Water, BA, and S sand were removed from the regression model because the tolerance limit was less than 0.1. The regression model was statistically significant (F = 73.336, *p* < 0.05), and the explanatory power of the regression model was approximately 98.7% (modified R squared was 97.3%) (R^2^ = 0.987, adjR^2^ = 0.973). Because the Durbin–Watson statistic was close to 2 (2.364), there was no problem with the assumption of the independence of residuals. In addition, the variance inflation factor (VIF) was less than 10 for all instances; therefore, there was no multicollinearity problem. As a result of the significance test of the regression coefficient, cement (=−0.242, *p* < 0.05), PRA (=−0.502, *p* < 0.01), and C sand (=−0.532, *p* < 0.01) all had a significant negative effect on carbonation depth, and RRA (=0.411, *p* < 0.01) had a significant positive effect. In other words, it was found that the higher the cement, PRA, and C Sand, the lower the depth of accelerated carbonation value; moreover, the higher the RRA, the higher the depth of accelerated carbonation value. Comparing the sizes of the standardization coefficients, it was verified that the depth of accelerated carbonation was greatly affected, in the order of greatest to least effect, by C sand (=−0.532), PRA (=−0.502), RRA (=0.411), and Cement (=−0.242).

The non-standardized regression equation of cement, RRA, PRA, and C sand for the depth of accelerated carbonation is as follows:

(4) $$y=31.246-0.037x_{1}+0.007x_{2}+0.008x_{3}+0.007x_{4}$$

where $x_{1}$, $x_{2}$, $x_{3}$, and $x_{4}$ represent cement, RRA, PRA, and C sand of the regression, respectively, and y describes the value of the depth of accelerated carbonation.

Pearson’s correlation analysis was conducted to analyze the correlation between the mechanical properties of concrete such as compressive strength, flexural strength, modulus of elasticity, the depth of accelerated carbonation, and raw materials, and the results are shown in Table 9. Statistically, water, cement, and BA had no correlation with compressive strength, flexural strength, and modulus of elastic (*p* > 0.05). In contrast, C sand had a significant negative correlation with compression strength, −0.710; flexural strength, −0.706; and modulus of elastic, −0.806 (*p* < 0.05). In addition, the correlation coefficients of general sand with compression strength, flexural strength, and modulus of elasticity were 0.727, 0.723, and 0.814, respectively, indicating a significant positive correlation (*p* < 0.05). That is, it was found that the concrete mixing ratio of water, cement, and basalt did not significantly affect the compressive strength, flexural strength, and elastic modulus of concrete only for the mixing ratio presented in this paper. For the depth of accelerated carbonation, water, cement, BA, S sand, and C sand were not statistically significant (*p* > 0.05), whereas RRA and PRA were found to be statistically significant (*p* < 0.05). The correlation coefficient of RRA and PRA was 0.675 and −0.724, respectively. It was found that when the proportion of RRA increased in the mixing concrete, the depth of accelerated carbonation increased, and when the proportion of PRA increased in the mixing concrete, the depth of accelerated carbonation decreased. For PRA, porosity was the only mechanical property that was statistically significant (*p* < 0.05). Considering that the correlation coefficient between porosity and PRA was −0.759, the porosity of concrete decreased as the proportion of PRA increased.

## 4. Conclusions

This study analyzed changes in the mechanical properties of concrete using the type of recycled aggregate, the type of fine aggregate, and the unit cement content as variables for the application of porous basalt-based recycled Jeju aggregate in the field. The findings are as follows.

1. The change in slump based on the replacement of the improved basalt-based recycled aggregate in the properties of non-hardened concrete was not significant. The slump increased as the fineness modulus of the fine aggregates decreased, and the unit cement content increased. Furthermore, the air content increased considerably when sea sand was used.
2. Regarding the strength properties of concrete, there was no major deterioration in quality due to the use of basalt-based recycled aggregate in concrete with a strength of 30 MPa at 28 days of age. The compressive strength of the concrete increased owing to the quality improvement of the basalt-based recycled aggregate and the even particle size distribution of the fine aggregates. The maximum compressive strength of concrete using recycled aggregate was 33.6 MPa, whereas the compressive strength of concrete using natural aggregate was 29.5 MPa for the concrete of 28 days of age.
3. The modulus of elasticity of concrete increased slightly due to the increased concrete’s fillability when the concrete was mixed with improved basalt-based recycled aggregates. The highest modulus of elasticity of 23.1 GPa was observed when uniform fine aggregates were used at 28 days age. The change in the modulus of elasticity using fine aggregates had a greater effect than the use of recycled aggregates.
4. The depth of accelerated carbonation of concrete decreased as the concrete matrix density increased when the improved basalt-based recycled aggregate was used. However, when RRA was used, the depth of carbonation increased rapidly with age. The depth of carbonation was large when fine aggregates of uneven grain size were used because dense concrete could not be formed.
5. The concrete pores increased in proportion to the pore content of the basalt and recycled aggregates. Pore content of 0.3 mm or larger decreased when improved basalt-based recycled aggregates and fine aggregates of even particle size were used. The decrease in pore content was directly related for the improvement of carbonation resistance and modulus of elasticity. Using recycled aggregates can be expected to increase the freeze–thaw resistance because the pore spacing factor was 200 μm or less and the specific surface area of the pore was close to 25 mm^2^/mm^3^.
6. In multiple regression and correlation analyses, it was found that the quality improvement of the fine and recycled aggregates improved the depth of carbonation resistance and the mechanical properties and durability index of concrete, respectively.
7. The recycled aggregate used in this experiment did not meet the domestic recycled aggregate standard of 3% or less. However, there was no deterioration in quality even with a unit cement content of 300 kg/m^3^ at a concrete mixing strength of 30 MPa due to the improvement in quality. Compared to basalt—a local natural aggregate—the improved recycled aggregate did not exhibit many differences in concrete quality. Consequently, it would be necessary to select a fine aggregate of good quality in the consideration of concrete quality and economic feasibility. Although additional verification of the mechanical properties and durability, such as dry shrinkage of recycled aggregate concrete, is required, its use in structural concrete products of 40 MPa or less was confirmed. It is believed that, with continued research, recycled aggregates mixed with valid industrial byproducts can be used as an effective substitute for basalt natural aggregates.

## Figures and Tables

**Table 1:** Experimental plan.

Coarse Aggregate Type	Fine Aggregate Type	Unit Cement Content (kg/m^3^)
Basalt (BA)	Sea sand (S)	300
Raw recycled aggregate (RRA)	Crushed sand (C)	330
Improved recycled aggregate (PRA)	Sea sand and crushed sand (SC)	360

**Table 2:** Mix proportion of the concrete.

	w/c	s/a	Unit Weight (kg/m^3^)							
			Water	Cement	Coarse Aggregate			Fine Aggregate		Total
					BA	RRA	PRA	S Sand	C Sand	
BA-S-300	0.50	0.40	150.0	300.0	1100.9	0.0	0.0	709.9	0.0	2260.8
BA-C-330	0.50	0.40	165.0	330.0	1062.6	0.0	0.0	0.0	671.9	2229.4
BA-SC-360	0.50	0.40	180.0	360.0	1024.3	0.0	0.0	264.2	388.6	2217.0
RRA-S-330	0.50	0.40	165.0	330.0	531.3	449.0	0.0	685.2	0.0	2160.5
RRA-C-360	0.50	0.40	180.0	360.0	512.1	432.8	0.0	0.0	647.6	2132.6
RRA-SC-300	0.50	0.40	150.0	300.0	550.4	465.2	0.0	284.0	417.6	2167.2
PRA-S-360	0.50	0.40	180.0	360.0	512.1	0.0	477.2	660.5	0.0	2189.8
PRA-C-300	0.50	0.40	150.0	300.0	550.4	0.0	512.9	0.0	696.1	2209.4
PRA-SC-330	0.50	0.40	165.0	330.0	531.3	0.0	495.0	274.1	403.1	2198.5

**Table 3:** Physical properties of the cement.

Density (g/cm^3^)	Fineness (cm^2^/g)	Setting Time (Hour)		Compressive Strength (MPa)			
		Initial	Final	3 Days	7 Days	28 Days	91 Days
3.15	3,818	4.5	7.15	23.0	29.3	42.5	52.5

**Table 4:** Chemical composition of the cement.

CaO (%)	SiO_2_ (%)	Al_2_O_3_ (%)	Fe_2_O_3_ (%)	MgO (%)	SO_3_ (%)	K_2_O (%)	Na_2_O_3_ (%)	Ig. Loss * (%)	Others (%)	Total (%)
62.44	21.12	4.40	3.19	3.10	1.19	0.32	0.26	3.36	0.62	100

* Ig. Loss: Loss of ignition.

**Table 5:** Physical properties of the aggregate.

Aggregate Type		G_max_ (mm)	Oven-Dry Density (g/cm^3^)	Absorption (%)	Fineness Modulus
Coarse aggregate	BA	25	2.60	3.16	6.90
	RRA	20	2.20	8.63	6.45
	PRA	20	2.43	5.19	6.45
Fine aggregate	S sand	5	2.52	1.45	1.61
	C sand	5	2.47	1.75	2.55
	SC sand	5	2.49	1.63	2.17

**Table 6:** Chemical composition of the aggregates.

Type	CaO (%)	SiO_2_ (%)	Al_2_O_3_ (%)	Fe_2_O_3_ (%)	MgO (%)	Na_2_O_3_ (%)	TiO_2_ (%)	K_2_O (%)	Others (%)	Total (%)
S sand	2.9	70.4	13.8	4.7	2.75	2.32	1.8	0.89	0.44	100
C sand	0.9	80.5	7.6	3.8	2.25	0.87	0.81	2.99	0.58	100
BA	9.01	48.36	13.95	11.88	9.02	2.92	2.02	2.18	0.66	100
RRA	31.4	36.2	9.86	7.84	5.97	1.77	1.53	1.81	3.62	100
PRA	16.5	45.3	12.13	10.08	7.66	2.41	1.89	1.91	2.12	100

**Table 7:** Independent and dependent variables and their source.

Category	Variable	
Independent Variable	Water	X1
	Cement	X2
	BA	X3
	RRA	X4
	PRA	X5
	S sand	X6
	C sand	X7
Dependent Variable	Depth of accelerated carbonation	Y3

**Table 8:** Multiple regression analysis for the depth of carbonation.

Dependent Variable	Independent Variable	B	S.E	*β*	t	*p*	VIF
Depth of carbonation	Constant	31.246	2.97	-	−10.520 ***	<0.001	-
	Water (kg/m^3^)	-	-	-	-	-	-
	Cement (kg/m^3^)	−0.037	0.009	−0.242	−4.160 *	0.014	1.003
	Basalt (kg/m^3^)	-	-	-	-	-	-
	RRA (kg/m^3^)	0.007	0.001	0.411	6.142 **	0.004	1.334
	PRA (kg/m^3^)	−0.008	0.001	−0.502	−7.483 **	0.002	1.336
	S sand (kg/m^3^)	-	-	-	-	-	-
	C sand (kg/m^3^)	−0.007	0.001	−0.532	−9.156 **	0.001	1.003
F = 73.336 (*p* < 0.05), R^2^ = 0.987, adjR^2^ = 0.973, D-W = 2.364							

* *p* < 0.05; ** *p* < 0.01; *** *p* < 0.001.

**Table 9:** Correlation analysis between the raw materials and mechanical properties.

	Water	Cement	BA	RRA	PRA	S Sand	C Sand
Water (kg/m^3^)							
Cement (kg/m^3^)	1.000 ** (0.000)						
Basalt (kg/m^3^)	−0.083 (0.832)	−0.083 (0.832)					
RRA (kg/m^3^)	−0.021 (0.958)	−0.021 (0.958)	−0.496 (0.174)				
PRA (kg/m^3^)	−0.021 (0.958)	−0.021 (0.958)	−0.496 (0.174)	−0.499 (0.171)			
S sand (kg/m^3^)	−0.033 (0.932)	−0.033 (0.932)	0.028 (0.943)	0.017 (0.966)	−0.041 (0.918)		
C sand (kg/m^3^)	−0.038 (0.922)	−0.038 (0.922)	−0.022 (0.955)	−0.015 (0.969)	0.042 (0.915)	−0.997 ** (0.000)	
Compressive strength (MPa)	−0.231 (0.549)	−0.231 (0.549)	−0.080 (0.838)	−0.416 (0.265)	0.526 (0.146)	−0.710 * (0.032)	0.727 * (0.027)
Flexural strength (MPa)	−0.246 (0.524)	−0.246 (0.524)	−0.048 (0.902)	−0.436 (0.240)	0.515 (0.156)	−0.706 * (0.034)	0.723 * (0.028)
Modulus of elasticity (GPa)	−0.108 (0.781)	−0.108 (0.781)	−0.206 (0.602)	−0.206 (0.595)	0.423 (0.257)	−0.806 ** (0.009)	0.814 ** (0.008)
Depth of accelerated carbonation (mm)	−0.219 (0.570)	−0.219 (0.570)	0.076 (0.845)	0.675 * (0.046)	−0.724 * (0.027)	0.566 (0.112)	−0.550 (0.125)
Porosity (%)	−0.230 (0.551)	−0.230 (0.551)	0.419 (0.261)	0.367 (0.332)	−0.759 * (0.018)	0.444 (0.231)	−0.428 (0.251)

(1) Numbers in ( ) indicate significance probability; (2) * *p* < 0.05, ** *p* < 0.01.

## Data Availability

Not applicable.
